# Cardiopulmonary bypass time, inflammatory response, and acute kidney injury in cardiac surgery: a retrospective study

**DOI:** 10.1590/1806-9282.20260819

**Published:** 2026-10-30

**Authors:** Yasemin Hacanlı, Murat Ziya Bağiş, Mahmut Padak, Ömer Göç, Kadir Egi, Mehmet Burak Coşkun

**Affiliations:** 1 Harran University, Faculty of Health Sciences, Department of Perfusion – Şanlıurfa, Türkiye.; 2 Harran University, Research and Application Hospital, Department of Cardiovascular Surgery – Şanlıurfa, Türkiye.; 3 Harran University, Vocational School of Health Services, Dialysis Programme – Şanlıurfa, Türkiye.; 4 Ministry of Health, Ordu State Hospital, Department of Cardiovascular Surgery – Ordu, Türkiye,

**Keywords:** Cardiopulmonary bypass, Acute kidney injury, Cardiac surgical procedures, Inflammation, Biomarkers

## Abstract

**OBJECTIVE:**

The aim of this study was to evaluate changes in inflammatory markers after cardiac surgery with cardiopulmonary bypass and assess the discriminatory ability of aortic cross-clamp time for postoperative acute kidney injury.

**METHODS:**

This retrospective single-center study included 109 patients undergoing cardiopulmonary bypass-assisted cardiac surgery between January 2024 and March 2025. Preoperative and postoperative (30–36 h) creatinine, urea, albumin, C-reactive protein, neutrophil, and lymphocyte levels were evaluated. Acute kidney injury was defined using the serum creatinine component of KDIGO criteria. C-reactive protein/albumin ratio, C-reactive protein/lymphocyte ratio, neutrophil/lymphocyte ratio, and albumin×lymphocyte/C-reactive protein (CALLY) were calculated. Given the limited number of acute kidney injury cases, analyses were exploratory. Aortic cross-clamp time was assessed by receiver operating characteristic analysis.

**RESULTS:**

Postoperative neutrophil, lymphocyte, and albumin levels differed significantly from preoperative values (all false discovery rate-corrected p<0.001). C-reactive protein, C-reactive protein/albumin ratio, C-reactive protein/lymphocyte ratio, neutrophil/lymphocyte ratio, and CALLY levels increased significantly (all false discovery rate-corrected p<0.001). Acute kidney injury developed in 15 patients and was more common among non-survivors (10.1 vs. 50.0%; p=0.006; false discovery rate-corrected p=0.022). Postoperative creatinine levels were higher in non-survivors (false discovery rate-corrected p=0.011). Aortic cross-clamp time showed limited discrimination for Acute kidney injury (area under curve=0.621). At the 52-min cutoff, sensitivity was 66.7%, and specificity was 60.2%.

**CONCLUSION:**

Cardiopulmonary bypass-associated cardiac surgery was accompanied by significant inflammatory changes. Aortic cross-clamp time showed limited discriminatory ability and should be considered an exploratory risk indicator rather than a standalone predictor of acute kidney injury.

## INTRODUCTION

Cardiac surgery includes various cardiovascular procedures^1^. Cardiopulmonary bypass (CPB) activates inflammation and coagulation, contributing to cardiac surgery-associated acute kidney injury (CSA-AKI), which increases morbidity, mortality, and hospital stay^2^. Prediction is challenging due to its complex pathophysiology^3^. Serum creatinine (Scr) is the most commonly used kidney marker; KDIGO defines AKI as an increase of ≥0.3 mg/dL within 48 h or ≥1.5-fold from baseline within 7 days^2^. Clinical risk models, such as the Cleveland Clinic and Mehta scores, identify high-risk patients using perioperative variables^4,5^. The severity and duration of AKI are associated with poor clinical outcomes^6^ and increased mortality^7^, highlighting the need for early identification and prevention. Additionally, "renal angina" aims to enable early AKI detection by combining susceptibility factors with early signs of renal stress^8^. In addition, neutrophil gelatinase-associated lipocalin (NGAL)^9^ kidney injury molecule-1 (KIM-1)^10^ and tissue metalloproteinase inhibitor-2 combined with insulin-like growth factor-binding protein-7 (TIMP-2×IGFBP7)^11^, it has been reported that new biomarkers such as these are capable of detecting kidney injury at an earlier stage than traditional creatinine-based criteria^9,10,12^.

The CALLY [Albumin×Lymphocyte/C-reactive protein (CRP)] index, comprising albumin, lymphocyte count, and CRP, has emerged as a marker of mortality outcomes^13^. NLR (neutrophil/lymphocyte ratio) and CLR (CRP/lymphocyte ratio) have also been associated with poor cardiovascular prognosis^14^.

In this retrospective study, we aimed to investigate the effect of CPB duration on biochemical parameters and inflammatory indices affecting renal function in preoperative and postoperative blood samples from patients diagnosed with heart disease and who underwent surgery using the CPB technique.

## METHODS

### Ethical approval

This study was conducted in accordance with the approval of the Harran University Clinical Research Ethics Committee (June 16, 2025; Decision No.: HRÜ/25.11.15) and the principles of the Declaration of Helsinki. Written informed consent was waived by the ethics committee due to the retrospective design, and patient data were anonymized before analysis.

### Inclusion and exclusion criteria

Cardiovascular surgery performed with CPB between January 1, 2024, and March 31, 2025 [coronary artery bypass grafting (CABG), heart valve surgeries, combined CABG+ aortic valve replacement (AVR), CABG+mitral valve replacement (MVR), atrial septal defect (ASD), and ventricular septal defect (VSD) repair] were evaluated. A total of 67 patients were excluded from the study due to pediatric status, emergency surgery, a history of dialysis, reoperation, the presence of a hematologic disease or malignancy, or incomplete preoperative/postoperative biochemical or clinical data. 109 patients were included in the study.

### Data collection and parameters evaluated

Patients’ biochemical parameters were assessed using the final routine preoperative laboratory results and postoperative data obtained 30–36 h after surgery. Baseline creatinine was the final preoperative measurement. Due to incomplete urine output data, AKI was assessed using the serum creatinine component of KDIGO criteria. The parameters examined included creatinine (Cr), albumin, standard CRP, and neutrophil and lymphocyte levels. Composite indices, including the CRP/albumin ratio (CAR), CLR, NLR, and CALLY, were also calculated, and their associations with AKI were investigated. Receiver operating characteristic (ROC) analysis assessed aortic cross-clamp time (ACCT), and univariable logistic regression assessed its association with postoperative AKI.

### Statistical analysis

Analyses were performed using IBM SPSS Statistics 27.0. Normality was assessed by the Shapiro-Wilk test and visual methods. Continuous variables were reported as mean±standard deviation or median (Q1–Q3), and categorical variables as n (%). CAR, CLR, and NLR were calculated as CRP/albumin, the CALLY index was calculated as [albumin (g/dL)×lymphocyte count (10^3^/μL)]/[CRP (mg/dL)×10]. Paired comparisons used the paired t-test or Wilcoxon signed-rank test, while survivor–non-survivor comparisons used the independent t-test or Mann-Whitney U test, as appropriate. Categorical variables were analyzed using Fisher's exact test. Missing values were not imputed. The Benjamini-Hochberg false discovery rate (FDR) method adjusted multiple comparisons; FDR-adjusted p<0.05 was considered significant. The sample included 109 consecutive patients meeting the inclusion criteria during the study period. No prior sample size or power analysis was performed. As AKI occurred in only 15 patients, AKI-related analyses were considered exploratory. ACCT's ability to distinguish AKI was assessed by ROC analysis, with the optimal cutoff determined using the Youden index. The association between ACCT and AKI was assessed by univariate binary logistic regression, with ORs and 95%CIs reported. All tests were two-sided.

## RESULTS

A total of 109 patients were included in the study. The mean age of the patients was 58.86±10.90 years; 67.0% were male, and 33.0% were female. Mean body surface area (BSA): 1.86±0.18, flow: 4467.63±430.21 L/min/m^2^, ACCT: 56.04±22.87 min, and total bypass time: 89.00±28.73 min. The percentage of patients with a family history was 44.0%, the percentage of patients who smoked was 54.1%, and the percentage of patients who consumed alcohol was 1.8%.

Postoperative neutrophil levels were statistically significantly higher than preoperative values (p<0.001), while lymphocyte and albumin levels were statistically significantly lower (p<0.001). All three of these differences remained statistically significant even after Benjamini-Hochberg FDR correction (all FDR-corrected p<0.001, Table 1).

**Table 1:** Preoperative and postoperative comparison of hematological markers.

Parameter	Preoperative Mean±SD	Postoperative Mean±SD	p^a^
Neutrophil (e^3^/μL)	5.50±2.23	10.77±2.91	**<0.001**
Lymphocyte (e^3^/μL)	2.28±0.81	1.32±1.15	**<0.001**
Albumin (g/dL)	4.12±0.68	3.12±0.69	**<0.001**

a Paired-samples t-test; SD: standard deviation. Bold values indicate statistically significant differences (p<0.05).

Of the 109 patients included in the study, 15 developed acute kidney injury. Postoperative Cr levels were significantly higher in non-survivors than in survivors (FDR-adjusted p=0.011). The incidence of AKI and the requirement for dialysis were also significantly higher in the non-survivor group (both FDR-adjusted p=0.022).

In the paired preoperative and postoperative comparisons, CRP, CAR, CLR, NLR, and CALLY values increased significantly after surgery, and all differences remained significant after Benjamini-Hochberg FDR correction (all FDR-adjusted p<0.001, Table 2).

**Table 2:** Comparison of renal function and posmiddleerative outcomes between non-survivors and survivor patients.

Parameter	Non-survivors	Survivors	Test	FDR adjusted p
Preoperative urea (mg/dL)	46.01 (39.06–54.04) [n=14]	35.68 (29.96–48.15) [n=95]	Mann-Whitney U	0.064
Posmiddleerative urea (mg/dL)	59.82 (33.17–95.23) [n=10]	36.38 (32.10–49.22) [n=99]	Mann-Whitney U	0.139
ΔUrea (mg/dL)	13.91 (-5.89–28.84) [n=10]	2.14 (-6.42–10.70) [n=99]	Mann-Whitney U	0.278
Preoperative creatinine (mg/dL)	0.98 (0.79–1.16) [n=14]	0.85 (0.73–1.00) [n=95]	Mann-Whitney U	0.278
Posmiddleerative creatinine (mg/dL)	1.30 (0.96–2.07) [n=10]	0.81 (0.67–0.95) [n=99]	Mann-Whitney U	**0.011**
ΔCreatinine (mg/dL)	0.36 (-0.06–0.78) [n=10]	-0.05 (-0.14–0.07) [n=99]	Mann-Whitney U	0.057
AKI ratio	5/10 (50.0)	10/99 (10.1)	Fisher exact	**0.022**
Dialysis rate	8/14 (57.1)	19/95 (20.0)	Fisher exact	**0.022**
Hospital stay	8.50 (5.25–13.25) [n=14]	13.00 (10.00–15.00) [n=95]	Mann-Whitney U	0.057
Cross-clamp ≥52 min	8/14 (57.1)	41/95 (43.2)	Fisher exact	0.394
Total perfusion time	100.00 (83.00–110.50) [n=14]	83.50 (70.00–96.00) [n=95]	Mann-Whitney U	0.064
Parameter	Preoperative Median (Q1–Q3)	Posmiddleerative Median (Q1–Q3)	Test	FDR adjusted p
CRP (mg/L)	1.11 (0.47–2.30)	16.09 (11.80–20.56)	Wilcoxon testi	**<0.001**
CAR	0.23 (0.10–0.51)	5.51 (3.79–6.56)	Wilcoxon testi	**<0.001**
CLR	0.55 (0.24–1.35)	10.35 (7.02–14.70)	Wilcoxon testi	**<0.001**
NLR	2.32 (1.69–3.22)	7.99 (5.16–10.04)	Wilcoxon testi	**<0.001**
CALLY	0.11 (0.04–0.29)	3.87 (2.48–4.80)	Wilcoxon testi	**<0.001**

AKI: acute kidney injury; CRP: C-reactive protein; CAR: CRP/albumin ratio; CLR: CRP/lymphocyte ratio; NLR: neutrophil/lymphocyte ratio; CALLY: Albumin×Lymphocyte/CRP; FDR: false discovery rate. Bold values indicate statistically significant differences (p<0.05).

In univariable logistic regression, each 10-min increase in ACCT was associated with higher odds of AKI, but this was not statistically significant (OR 1.14, 95%CI 0.91–1.43; p=0.252). ACCT ≥52 min showed approximately threefold higher odds of AKI, although this was also not statistically significant (OR 3.03, 95%CI 0.95–9.62; p=0.060).

Given the limited number of AKI events, multivariable logistic regression was not performed because of the risk of model overfitting.

In the ROC analysis, the ability of ACCT to distinguish the development of AKI was calculated as AUC=0.621. This value indicates that ACCT provides weak to moderate discrimination in predicting AKI in the current dataset. Based on the cutoff value, the optimal threshold was determined to be 52 min. At this threshold, sensitivity was 66.7%, and specificity was 60.2%.

Patients were divided into two groups based on the 52-min cutoff value obtained from the ROC analysis: <52 min and ≥52 min. The AKI rate was 8.6% in the <52-min ACCT group and 22.2% in the ≥52-min ACCT group. Although the AKI rate was numerically higher in the ≥52-min ACCT group, the difference did not reach statistical significance (p=0.089). Postoperative Cr levels were also higher in the ≥52-min ACCT group; however, this difference was borderline significant (p=0.059, Figure 1).

**Figure 1:**
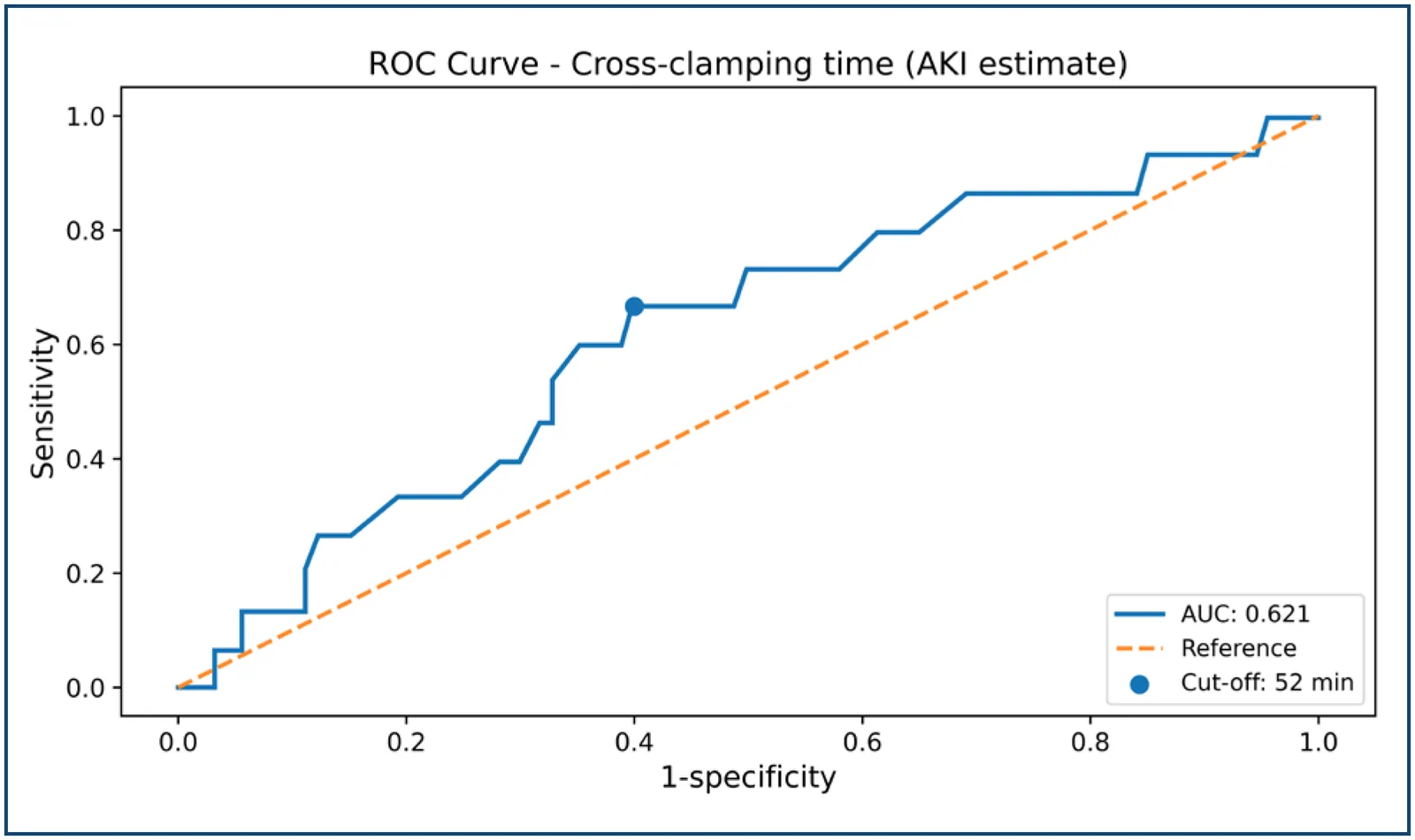
Receiver operating characteristic analysis: predicting acute kidney injury using cross-clamp time.

## DISCUSSION

Postoperative AKI was assessed using the serum creatinine component of the KDIGO criteria and defined as a ≥0.3 mg/dL or ≥50% (≥1.5-fold) increase from the preoperative baseline. Patients meeting either criterion were classified as having AKI.

In an analysis of 2,844 cardiac surgery patients, the development of AKI was found to be associated with high preoperative Scr, advanced age, and prolonged CPB time^15^. In a study comparing patient groups with long- and short-CPB durations, it was observed that an increase in ACCT was associated with higher Scr levels at 24 and 48 h and higher urea levels at 48 h, and that AKI developed^16^.

Blood urea nitrogen is widely used to assess cardiorenal status and predict cardiovascular outcomes^17^. Tolomeo et al. showed that urea levels and the urea/creatinine ratio predict acute heart failure^18^, while perfusion time and age have been reported as risk factors for AKI^19^. In our study, postoperative urea and creatinine levels increased significantly, supporting increased renal stress after surgery.

Also, albumin levels decreased significantly after surgery. This finding is consistent with previous studies attributing this change to systemic inflammation, capillary leakage, and CPB-associated hemodilution^20^.

CAR reflects the balance between CRP and albumin, whereas CALLY additionally incorporates lymphocyte count, providing information on inflammatory, immune, and nutritional status^13^. Previous studies have shown that lower CALLY values are associated with higher mortality^13,21^, whereas elevated CAR has been linked to poorer cardiovascular outcomes^22^.

NLR and CLR have been associated with poor cardiovascular prognosis^14,23^. Consistent with these findings, all inflammatory indices increased significantly after CPB in our study, suggesting their potential value in postoperative risk stratification. Furthermore, the more pronounced increase in these inflammatory indices in patients who developed AKI and experienced mortality suggests that these parameters may be helpful in postoperative risk stratification.

AKI was more frequent among non-survivors. ACCT showed limited predictive performance; although ACCT ≥52 min was associated with approximately threefold higher odds of AKI, this association was not statistically significant. Our cohort was not restricted to low-risk elective CABG or isolated valve procedures; thus, mortality should be interpreted in the context of its heterogeneous clinical characteristics. The association between AKI and mortality should be interpreted cautiously, as the retrospective design cannot establish causality. Larger prospective studies with multivariable adjustment are needed.

Although ACCT has been associated with postoperative AKI, current evidence suggests that it should be interpreted as part of multifactorial risk models rather than an independent predictor^24,25^. In our study, ACCT showed limited discriminatory ability (AUC=0.621). At the optimal cutoff of 52 min, sensitivity was 66.7%, and specificity was 60.2%. These findings indicate that ACCT alone has limited clinical utility and should not be used as a standalone tool for postoperative AKI risk stratification.

The in-hospital mortality observed in our cohort was higher than that commonly reported for elective isolated CABG or valve surgery populations. This difference likely reflects the characteristics of our study population, which consisted of an unselected tertiary-care cardiac surgery cohort rather than a low-risk elective population. Moreover, the association between postoperative AKI and mortality should be interpreted cautiously. Because of the retrospective observational design, the present study cannot determine whether AKI independently contributes to mortality or simply reflects greater postoperative illness severity. Larger prospective studies with multivariable adjustment are required to clarify this relationship.

## LIMITATIONS

One of the major limitations of this study is the low number of patients who developed acute kidney injury. Although a total of 109 patients were included in the study, only 15 developed AKI. This limited the precision of the effect estimates and the statistical power of the study, and made it difficult to perform reliable multivariate adjustments and meaningful subgroup analyses. For this reason, findings associated with AKI should be considered exploratory and should not be interpreted as evidence of independent or causal relationships. These findings need to be validated by multicenter studies with larger sample sizes and sufficient statistical power.

## CONCLUSION

This study demonstrated significant changes in hematological and inflammatory parameters following cardiac surgery. AKI developed in 15 patients and was more common among non-survivors, who also had a higher rate of dialysis requirement. The discriminatory performance of ACCT for AKI was limited. Due to the limited number of AKI events, multivariable analysis could not be performed. Therefore, these findings should be interpreted as exploratory and confirmed in larger, multicenter studies.

## Data Availability

The datasets generated and/or analyzed during the current study are available from the corresponding author upon reasonable request.
